# Efficacy of the In2Care® auto-dissemination device for reducing dengue transmission: study protocol for a parallel, two-armed cluster randomised trial in the Philippines

**DOI:** 10.1186/s13063-019-3376-6

**Published:** 2019-05-14

**Authors:** Ferdinand Salazar, Jason Angeles, Ava Kristy Sy, Marianette T. Inobaya, Ariza Aguila, Tom Toner, Michael J. Bangs, Edward Thomsen, Richard E. Paul

**Affiliations:** 1 Department of Medical Entomology, Research Institute for Tropical Medicine, Filinvest City Alabang, Muntinlupa City, Philippines; 2 Department of Virology, Research Institute for Tropical Medicine, Filinvest City Alabang, Muntinlupa City, Philippines; 3 Department of Epidemiology and Biostatistics, Research Institute for Tropical Medicine, Filinvest City Alabang, Muntinlupa City, Philippines; 4 Liverpool School of Tropical Medicine, Liverpool, UK; 5 PT Freeport Indonesia/International SOS, Kuala Kencana, Indonesia; 6 Kasetsart University, Bangkok, Thailand; 7 Functional Genetics of Infectious Diseases Unit, Institut Pasteur, Paris, France; 8 Génomique évolutive, modélisation et santé UMR 2000, Centre National de la Recherche Scientifique (CNRS), Paris, France

**Keywords:** Auto-dissemination, Pyriproxyfen, *Beauvaria*, Dengue, *Aedes aegypti*

## Abstract

**Background:**

Mosquito-borne viruses are imposing an ever increasing health burden worldwide. In addition to the recent Zika and chikungunya virus epidemics, dengue viruses have become the fastest growing problem with a 40-fold increase in the number of reported cases over the past five decades. Current mosquito control techniques involving larval source reduction, larviciding, and space spray of adulticides are costly, laborious, and of debatable efficacy. There remains an urgent need for the development of intervention methods that can be reasonably implemented in the context of modern day urbanisation. Auto-dissemination (AD) of insecticide by adult mosquitoes offers a potentially practical and useful tool in an integrated vector control programme. Recently, an immediately employable AD device, the In2Care® mosquito trap, has been commercialised and shows promise as an effective tool. However, there remains a lack of demonstration of epidemiological efficacy.

**Methods/design:**

This trial aims to assess the extent to which implementation of In2Care® mosquito traps can reduce vector *Aedes* (*Stegomyia*) spp. adult mosquito densities and dengue virus transmission as measured by sequential sero-conversion rates in children 6–16 years of age in a dengue endemic location: Lipa City, Philippines. To achieve this, we will carry out a parallel, two-armed cluster randomised trial evaluating AD efficacy for reducing the incidence of dengue over a 2-year period with 4 consecutive months of vector control during peak dengue transmission each year.

**Discussion:**

For decades, it has been commonly accepted that an integrated approach to mosquito control is required. The World Health Organization (WHO) Global Strategic Framework for Integrated Vector Management recommends a range of interventions, in combination, to increase control impact to reduce transmission. This efficacy trial of the first commercial product using the AD approach will be informative in assessing the general utility of AD in reducing not only adult vector densities but, more importantly, reducing the incidence of dengue. The AD technique may complement source reduction and larviciding campaigns by more efficiently targeting the most productive containers and those beyond human reach. If successful, this mosquito control strategy could prove an invaluable tool in the fight against urban mosquito vectors and a reduction in the burden of associated disease.

**Trial registration:**

ISRCTN44272773. Registered on 31 January 2019.

**Electronic supplementary material:**

The online version of this article (10.1186/s13063-019-3376-6) contains supplementary material, which is available to authorized users.

## Background

Mosquito-borne viruses of public health importance are imposing an ever increasing burden worldwide. In addition to the recent notable epidemics of West Nile, Zika, and chikungunya viruses, the dengue viruses in particular have become the fastest growing problem with a 40-fold increase globally in the number of reported cases over the past five decades affecting over 100 countries [1]. Currently, an estimated 400 million dengue virus (DENV) infections occur annually, of which only a quarter or less become symptomatic [2]. Of these, a small percentage manifest into more severe forms of the disease: dengue haemorrhagic fever and/or dengue shock syndrome and potentially death. The primary mosquito species responsible for DENV transmission is *Aedes aegypti* (L.), a species that has adapted to an urban habitat, proliferating in artificial water containers arising primarily from solid waste and frequent long-term water storage. Rates of urbanisation are increasing dramatically globally, particularly in regions of high socio-economic vulnerability, exacerbating an already over-stretched infrastructure and low public service capacity. The increasing frequency and amplitude of dengue epidemics bears testament to the scale of the threat and the urgent need to address these diseases in the context of a rapidly changing urban landscape [3].

Despite progress in the development of vaccines for preventing chikungunya, dengue, and Zika infections [4–6], no current candidates appear likely to have general application in endemic areas. Therefore, it is generally agreed that an integrated approach to control, including a significant role for vector abatement, will be required [7, 8]. For over 100 years, the most common dengue control strategy has been source (larval habitat) reduction and focal insecticide treatment of mosquito larval habitats (so-called ‘breeding sites’). This approach helped to eliminate *Ae. aegypti* from 22 countries in the Americas during control campaigns in the 1950s and 1960s [9]. However, these activities required enormous human resources and costs to support, making them unsustainable in the long term. Today, the scale of continuing urban expansion abnegates such an approach. Current mosquito control techniques based on larval habitat source reduction, larviciding, and space spray are costly, laborious, and of debatable efficacy depending on the circumstances [10, 11]; therefore, there remains an urgent need for the development of intervention methods that could be reasonably implemented in the context of modern day urban environments and with some measure of possible sustainability. One of the major challenges in large, condensed urban settings is achieving sufficient coverage of aquatic habitats preferred by *Ae. aegypti* [12]. Identifying and treating all possible breeding sites manually is not a realistic solution even with considerable organised community support and resources.

One potential solution, originally proposed by Itoh et al. [13], is to exploit the ‘skip-oviposition’ behaviour of adult female *Ae. aegypti* to disseminate insecticide to natural larval habitats [14], in other words female mosquitoes exposed to a surface contaminated with insecticide subsequently spread it to other aquatic sites during oviposition [12, 15], a mechanism called auto-dissemination (AD). Insofar as the mosquitoes cannot “carry” substantial amounts of the particulate chemical matter on their tarsi and the insecticide must work in even large volumes of water, the insecticide must be potent at very minute (nanogram) concentrations. Furthermore, given the relatively indiscriminate dissemination of the insecticide, it must also be non-toxic to vertebrates and have a relatively narrow range of action against other aquatic invertebrates. Pyriproxyfen (PPF) ticks all the boxes [16]. This World Health Organization (WHO) approved pupacide can be safely used in drinking water and is recommended as part of conventional programmes against *Ae. aegypti* [17, 18]. It is a synthetic analogue of juvenile hormone which, at very low concentrations, prevents larval and pupal development as well as female fertility and male spermiogenesis [19, 20]. Currently it is applied as either granular or liquid formulations directly to water containers and drains in South America and South East Asia with documented success in reducing immature and adult mosquito numbers and has been associated with a reduction in dengue incidence [21, 22]. The potential of PPF application via AD has been demonstrated in a variety of small-scale trials in Peru and Italy [12, 15] and on a larger scale in the Amazon [23]. However, whilst entomological efficacy of PPF has been demonstrated when applied through AD, ground space spray, or container treatment, evidence of actual epidemiological impact (infection reduction) is generally inconclusive [24, 25].

Recently, a commercialised AD device, the In2Care® mosquito trap (In2Care® BV, Wageningen, Netherlands) [26], has been validated in field studies in the Grand Cayman, yielding pupal mortality rates of ~ 60% (unpublished data). The In2Care® trap attracts ovipositing *Ae. aegypti* females, which are then contaminated with a combination of PPF and an entomopathogenic fungus, *Beauvaria bassiana*, a United States Environmental Protection Agency approved agent that has been used in biological control of many agricultural pests for decades [27]. The lethal and pre-lethal (e.g. reduced feeding propensity) effects of the fungus have been described in *Anopheles* and *Aedes* species in which it takes several days to kill the adult insect, thereby allowing the mosquito to disseminate the PPF component [28, 29]. The fungal infection also interferes with DENV replication within the mosquito, thus having a profound effect on mosquito vectorial capacity (i.e. transmission efficacy) beyond that of shortening the adult mosquito lifespan [30]. The In2Care® mosquito trap thus uses an AD approach to disseminate a pupacide combined with a direct action, slow-acting entomopathogenic adulticide.

This study proposes to evaluate the epidemiological and entomological efficacy of the In2Care® trap in a dengue-endemic urban location in the Philippines.

## Methods/design

### Objectives

The primary objective of the trial is to assess the extent to which implementation of In2Care® mosquito traps can reduce *Ae. aegypti* adult mosquito densities and DENV sero-conversion rates (i.e. transmission) in children 6–16 years of age in a site of endemic dengue risk in the Philippines.

### Study type

The study is a parallel, two-armed cluster randomised trial (PCRT) evaluating the efficacy of the In2Care® mosquito trap for reducing incidence of dengue infection over a 2-year period using 4 consecutive months of treatment each year.

**Fig. 1:**
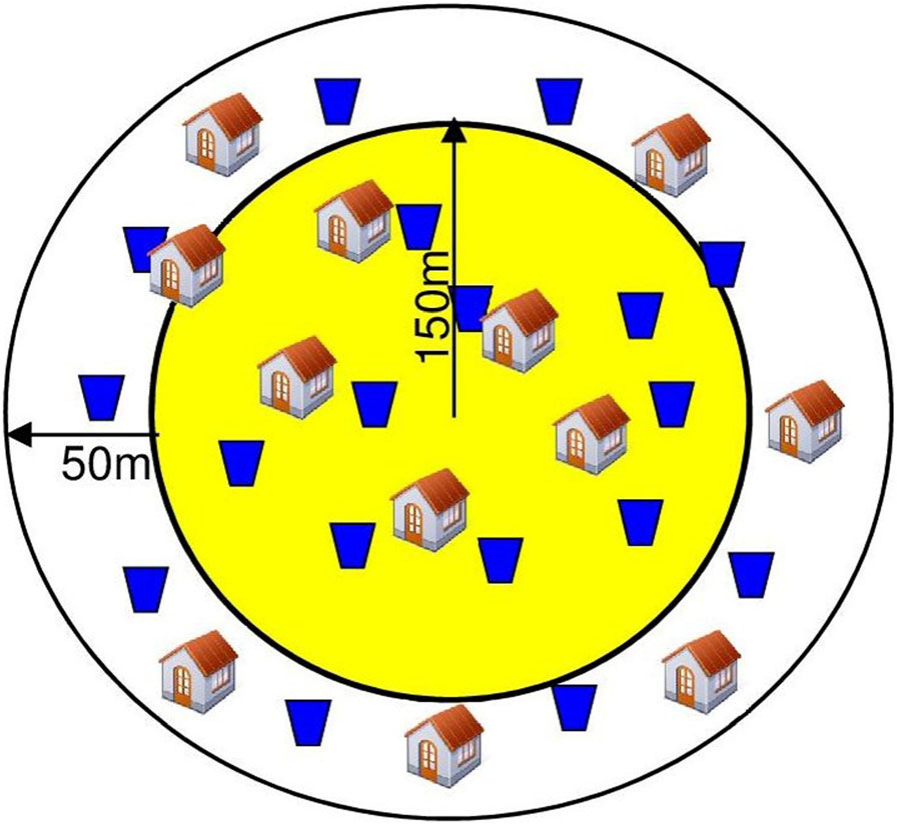
Intervention and sampling strategy. The yellow region includes recruited children for the sero-conversion study and the outer peripheral region is the buffer zone also treated with AD devices (blue jar icons) but without sero-conversion saliva sampling

Previous studies have estimated that, to account for the unpredictable spatio-temporal heterogeneity in dengue transmission, PCRTs are the most effective study design and that a larger number of clusters with fewer children per cluster provide the optimal strategy—in effect, a bet-hedging approach for a statistically sufficient coverage of an area [31]. In order to attempt to control for the spatial heterogeneity in dengue occurrence, 46 areas (hereafter referred to as “clusters”), each of approximately 200 m in radius, will be pre-selected. Twenty-three clusters will be assigned to the treatment arm with In2Care® traps and 23 to the control arm without traps. Cluster sites assigned to treatment versus control arms will be matched according to retrospective incidence of reported dengue clinical cases over the previous 5 years and human population density (See Stratification scheme below). Because of ‘edge effects’, where individuals near the periphery of the treatment area will be potentially subject to contact with mosquitoes coming from neighbouring non-treated areas, we will implement the “fried egg” design [32] whereby we will assess sero-conversion only in children resident at least 50 m from the treatment zone edge. Thus, whilst we will implement In2Care® traps throughout a 200 m radius circle, we will only consider sero-conversion in children within a 150 m radius (Fig. 1). One hundred schoolchildren (between 6 and 16 years of age) will be recruited for repeated sero-conversion observation within the inner circle of each of the 46 clusters (See Sample size calculations below). As the population density in clusters will vary, it is possible that there will not be sufficient children within a prescribed area. In this case, we will increase the circle radius to reach the required sample number.

Adult *Aedes* mosquito densities will be monitored in treatment and control sites by the use of Gravid *Aedes* Traps (GATs; Biogents AG, Regensburg, Germany) (Additional file 1). GATs are more efficient in capturing gravid *Ae. aegypti* than sticky ovitraps and are practical, low cost, and easily transportable devices, essential attributes in large-scale monitoring programmes [33].

### Stratification scheme

Clusters will be stratified according to historical dengue incidence and population density. In the Philippines, a *barangay* is the smallest administrative division in the Philippines (urban or rural), but which can be subdivided into *puroks* (zones) consisting of 20 to 50 households depending on the location and density of the households in an area. The previous 5 years of dengue incidence data at the *purok* administrative unit level will be acquired from the Philippines Integrated Disease Surveillance and Response (PIDSR) system to identify relative defined ‘hotspots’ and ‘coldspots’ of dengue activity during that period. This information will be entered into the Disease Data Management System (DDMS+) (see Data management below) along with information on geography, population density, and infrastructure at the *barangay* level. This will generate maps with which to choose clusters for the intervention study and assign clusters to either treatment or control groups according to broad similarities.

### Sample size calculations

The sample size is calculated using the following standard formula when considering proportions (incidence rate):

$$ n=2{\left(\mathrm{Za}+\mathrm{Z}1-\upbeta \right)}^2\ \mathrm{p}\left(1-\mathrm{p}\right)/{\left(\mathrm{p}0-\mathrm{p}1\right)}^2 $$

Where *n* is the required sample size per treatment arm (control or treatment), Zα and Z1–β are constants set by convention according to the accepted α error and whether a one-sided or two-sided effect, p0 is the proportion infected in control areas, and p1 the proportion infected in treatment areas (p = (p0+p1)/2).

Assuming a *p* < 0.05 as acceptable and a study with 90% power, the following constant values are: Zα = 1.96 (two-tailed); Z1–β = 1.2816.

The final sample size will depend on the dengue transmission rate in the study areas as estimated from the historical mean rate. Recent sero-conversion studies in Cebu City, Philippines, have observed a symptomatic incidence rate of 1.6 per 100 person-years (1.6%) with 7% subclinical infections in the general population, increasing to 7% symptomatic cases and 17% subclinical infections in children < 15 years old [34]. Historical annual incidence rates were estimated at between 11 and 22% [35]. Reported incidence rates in our study area (under administrative Region IV-A) are consistently amongst the highest in the country. To be conservative, however, we estimate an infection incidence of 3% (to include symptomatic and subclinical infections). For an efficacy of treatment of 50% (i.e. reduction in dengue), with 90% power, a minimum of 2054 children (aged 6–16 years old) will need to be recruited in treatment sites with an equivalent number in matched control sites and followed for 2 years. We factor in a drop-out of 250 children (~ 12.2%) per treatment and control arm (500 total) over the 2-year period. Thus, the total number of children to be recruited is 4600, distributed over 46 clusters of 100 children each.

### Setting (Fig. 2)

Dengue is endemic throughout much of the Philippines, and between 2008 and 2012 the country’s Department of Health (DoH) reported an annual average of 117,065 diagnosed (symptomatic) dengue cases, although the number is likely to be far higher because of significant under-reporting [36]. Region IV-A (Calabarzon) has consistently had a high burden of dengue and was one of three regions where the dengue vaccine (Dengvaxia®, Sanofi Pasteur) was implemented. According to an official DoH report, in 2018 the Calabarzon region was the area most affected by dengue, accounting for 19% of all national cases, while also showing an increase in case load of 40% in comparison with the same time period the previous year. Lipa City is a large city in the Calabarzon region and is located approximately 78 km south of Manila at an altitude of 312 m, covering an area of 209.40 km^2^ with a population of 332,386 people in 2015. The city is subdivided into 72 *barangay*s of varying human density (at an average of ~ 1800/km^2^). The average annual temperature is 28 °C (82 °F) and the peak dengue transmission season typically occurs during increased rains from June to December.

**Fig. 2:**
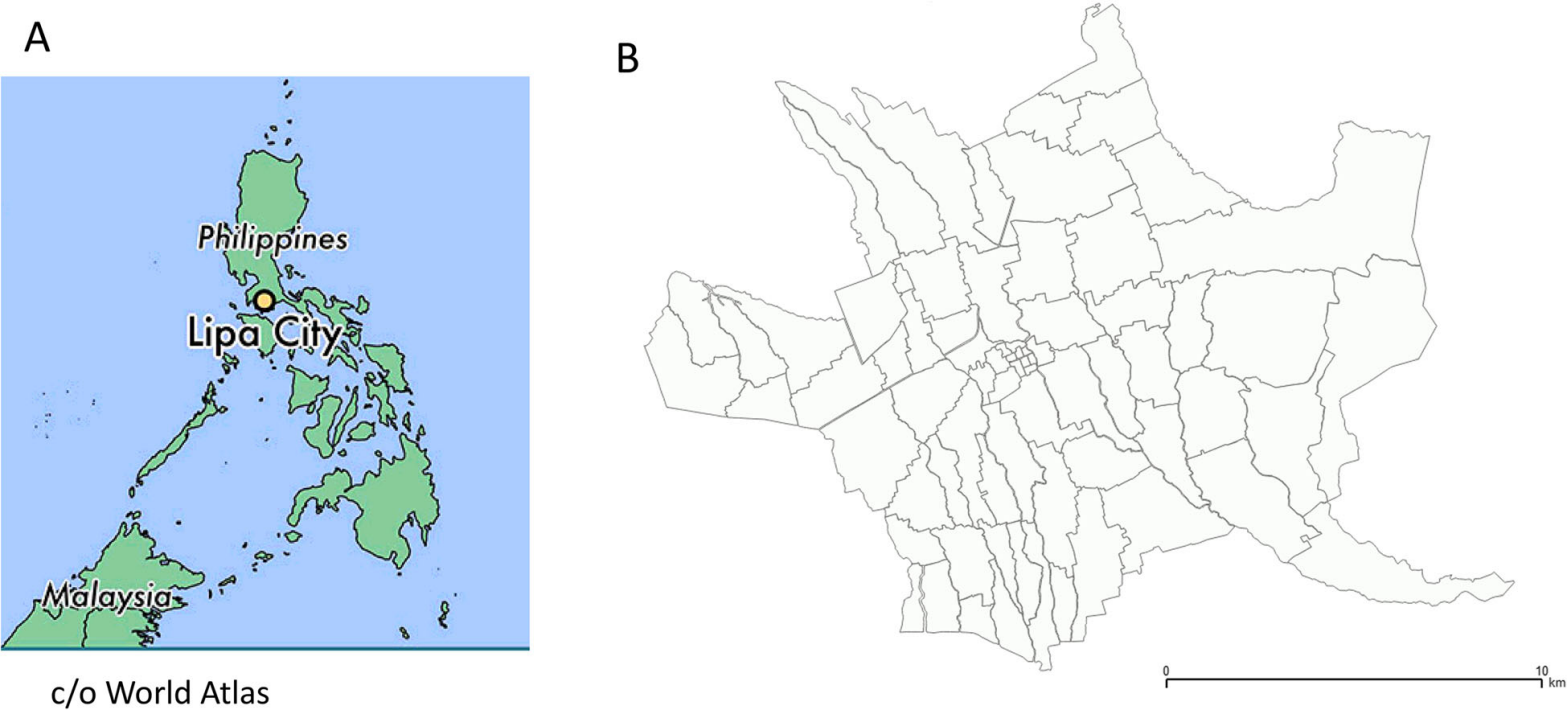
Maps of the study site. **a** The Philippines; **b** Lipa City *barangay*s

### Outcomes

The principle evaluation criterion is the epidemiological efficacy read out: dengue infection sero-conversion. Sero-conversion to dengue virus exposure will be measured by an enzyme-linked immunosorbent assay (ELISA) for immunoglobulin (Ig)G titres using repeated saliva samples taken sequentially during three cross-sectional studies at months 0, 3 (end of month 2), and 5 (end of month 4) during the intervention (In2Care® trap placement) period each year in both treatment and control sites. These time points are designated Pre-, Post1, and Post2 within each year.

The secondary evaluation criterion is the entomological read out. To measure the impact of the In2Care® traps on the adult mosquito population densities, 20 GATs will be placed in each of 10 randomly selected treatment and 10 control sites matched with intervention sites for historical dengue incidence. *Aedes* spp. (both *Ae. aegypti* and *Ae. albopictus*) adults will be counted and identified in the GATs once each week beginning 2 weeks before intervention, throughout the intervention period, and 2 weeks post-intervention. Traps will be placed both inside and outside 10 randomly selected houses within each site. Traps placed outside will be positioned against the wall of the house in an area with shelter from direct sunlight. Traps placed inside the house will be positioned in a suitably unobstructed area but where there is air movement.

### Study subject eligibility criteria

Inclusion criteria include: male and female individuals 6–16 years old; a parent/guardian giving informed consent for the child’s participation in the study; and, for children > 7 years old, required assent for his/her participation in the study and written assent from children 12–16 years old.

The exclusion criterion is a child with known concomitant pathology(ies) at the time of the consent as indicated by the parent/guardian

### Recruitment

In each *barangay* pre-selected to participate in the study, Research Institute for Tropical Medicine (RITM) team members will hold general information meetings on the project with the population in the presence of the community (*barangay*) health workers (BHWs) and local public health officials. Because the volunteers to be recruited are of school age, additional information meetings will be held in the local schools. Once clusters are identified and randomly assigned with appropriate stratification, the study team will perform household visits with the Lipa City public health staff and respective area BHWs. The visit will explain the study and study protocol in detail and request individual informed consent/assent for periodic saliva sampling and mosquito trap placement by each household head (In2Care® and/or GATs) in and around participant’s houses (See Ethics approval and consent to participate below).

Following signed consent, a short questionnaire pertaining to the participant will be completed and the individual assigned a unique identifier barcode. The questionnaire will be completed on paper including details on: name, address, age, sex, whether having received the Dengvaxia® vaccine (and if yes, how many injections) or not, and the attending school and year (grade). The individual’s barcode will be placed next to their name on the consent form and kept in a separate file. Subsequent samples will also be labelled with a unique barcode and linked back to the individual’s barcode in the questionnaire.

### Data confidentiality

During field investigations, a file with a name and address and corresponding barcode assigned to the subject identity will be used. This file will only be accessible to the field investigations team members who have direct contact with the participants. To ensure confidentiality, the file will be kept in an access-limited and secured (locked) cabinet. Upon completion of the field work, the address of the individual will be removed from the database, with irreversible anonymisation. Fieldwork will involve initial paper files that will then be input into an electronic file which will be accessible only to the field investigation team.

Study compliance will strictly adhere to the Republic Act No. 10173 (Philippines Data Privacy Act) stipulating full protection of an individual’s personal information by coding and secure data storage, with no dissemination of this information outside the study context. Participants can request their personal information to be removed and destroyed at any time during the study, which will also result in the withdrawal of the participant from the study.

### Intervention (Fig. 3)

Throughout the study period, local health authorities will continue with their routine government approved dengue control strategies. Our intervention study will in no way replace the current mosquito control methods in use.

**Fig. 3:**
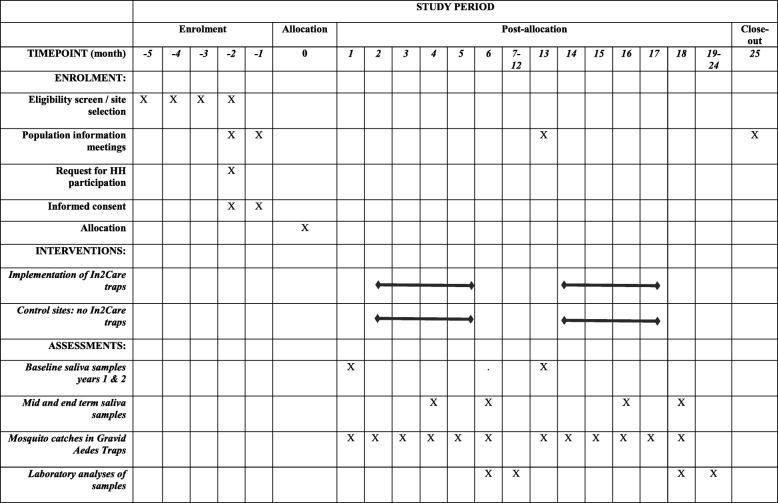
Time schedule of enrolment, interventions, and post-allocation data collections (based on SPIRIT 2013 figure [37]). HH Household

For the intervention study, 46 clusters will be selected each covering an area with at least 100 children. Mean child (age 6–16 years) densities in urban sites are 18/ha (/0.01 km^2^). Thus, an average sampling radius of 140 m (6.15 ha) would generate 100 children (excluding 10% outright refusal) to be recruited with an additional 50 m radius buffer zone for treatment but without saliva sampling. The area to be treated would thus be 113,000 m^2^ per cluster and would require 113 AD devices at a density of 1 trap/1000 m^2^ per treated cluster (23 study clusters to be treated). Before geolocalisation of historical dengue cases, however, we cannot predict which sites will be selected, nor their actual human densities, and thus the initial calculations are based on mean densities.

Treatment of areas with In2Care® devices will be carried out for 4 months each year and the active ingredient sachet (combination PPF + fungus) replaced every 6 weeks. Each In2Care® trap will be assembled (Additional file 2) and filled with 3.5–4.5 l of clean tap water. One pesticide-treated netting removed from the active ingredient sachet will then be placed on the floater and the remaining contents of the sachet tipped into the water. Each (10 × 18 cm) sealed aluminium refill sachet contains: 0.5 g In2Mix (containing 10% *Beauveria bassiana* strain GHA (Chemical Abstracts Service (CAS) No. 63428–82-0), 74.03% pyriproxyfen (CAS No. 95737–68-1), 0.97% pyriproxyfen impurity, and 15% insert silicon dioxide); one 5 × 49 cm gauze strip; and two yeast food supplement tablets of common brewer’s yeast (*Saccharomyces cerevisiae*). The tablets are added to the trap tap water to emit organic odours attractive for egg-laying mosquitoes.

In2Care® traps will be placed in open areas outside of the house. GATs will be placed inside and outside houses at designated locations. GATs will be assembled as per the manufacturer’s instructions and filled with water to which a handful of dry grass/hay will be added. A sticky strip is placed inside the GAT and used to capture entering mosquitoes (Additional file 3). The general state of the traps (In2Care® and GATs) will be assessed during the weekly GAT counts and water replenished where necessary. Likewise, the dried grass and water in the GATs will be replaced during the weekly survey. After 6 weeks, the netting, active ingredient, and yeast tablets from the sachet of the In2Care® mosquito trap will be replaced with a new sachet and new water. At the end of the 4-month treatment period, all mosquito traps will be removed, properly cleaned, and stored until the following year.

### Saliva sampling and biological analyses

A saliva sample will be taken by spitting into a sterile urine collection container, kept on ice, and transported to RITM for further processing. A 1-mL aliquot will be placed in a labelled Eppendorf® tube for storage at −20 °C. Sampling will occur just prior to, during (after 2 months of trap deployment), and at the end of the 4-month intervention period for 2 successive years. Sero-conversion testing will be performed using an in-house ELISA developed by Institut Pasteur [38] in duplicate on “paired” pre-/concurrent/post-samples from the same individual for each season. Although IgM titres will rise during a secondary DENV infection, a significant increase in IgM titres only systematically occurs in primary dengue infections and is relatively short lived; therefore, changes in IgG titres will be measured [38]. Exposure to dengue virus will produce an increase in IgG titre compared with the baseline titre in an individual, irrespective of whether they have been vaccinated or not or having been previously exposed to any DENV serotype (See Statistical analyses below). The technicians performing the ELISAs will have no prior knowledge whether the samples come from a treatment or control site. Upon study completion, analyses, and publication, all saliva samples will be destroyed and coding files erased.

### Specific data collection

For each participating child, information on their name, age, sex, address, *barangay*, cluster number, dengue vaccination status, school, class, and subsequent dengue ELISA titres will be recorded using a paper case report form that will be subsequently input into a study electronic database. Unique bar codes will be used as identifiers.

Data will be entered directly into DDMS (See Data management below) with 10% cross-checking.

Data will remain secured at the Entomology unit in RITM, Alabang, The Philippines. Statistical analysis will be performed in RITM.

After the final saliva sample has been taken and the data cross-checked for errors, the file linking the individual’s name and code will be destroyed.

### Benefits to the individual and the community

Study volunteers and their parents will be informed on their specific sero-conversion ELISA findings during an annual feedback meeting. Whilst not providing a definitive diagnostic for infection, at a minimum this will inform individuals whether they are sero-positive for dengue and whether they have had a primary or post-primary infection. Because there is an increased risk of severe disease in post-primary infections, this information could enable individuals to enhance their efforts for personal protection from mosquito bites. A major benefit of the study is directed to the entire community, and potentially the entire country and beyond, if there is evidence that the use of In2Care® mosquito traps can decrease the risk of dengue transmission.

### Data management

Emerging innovative information technologies, such as data management system software packages, can assist disease control programmes to better manage and analyse data, and thus make it easier to carry out routine surveillance, monitor interventions, and evaluate control programme performance. This will lead to better informed decision making and actions on the part of health authorities. The Liverpool School of Tropical Medicine have developed a multi-disease data management system (DDMS+) platform that facilitates the real-time monitoring and evaluation of interventions that are essential for understanding the progress, success, and challenges facing disease control operations [39]. DDMS+ is an open source data management software program that works as a modular, integrated system. Currently, DDMS+ has been successfully trialled for other vector-borne diseases in eight countries for malaria and one for leishmaniasis. The DDMS+ will be implemented for the dengue study and bring this state-of-the-art technology to the Philippines Department of Health through training exercises and workshops.

### Statistical analyses

#### Classification of dengue incidence as ‘hot’, ‘intermediate’, and ‘cold’ spots

Dengue incidence for the previous 5 years will be acquired at the *purok* level, and the midpoint of the *purok* used as the geographical coordinate representing the *purok*. Population density at the *barangay* level will be acquired. Hot/coldspot classification of dengue incidence rate will be achieved through analysis using Kulldorff’s scan statistic in SaTScan™ (version 9.1.1) (http://www.satscan.org/) [40]. A discrete Poisson model will be used to analyse the spatial distribution of dengue cases using all 5 years of incidence data, with year as the analysis time unit and population density as a covariate. Within SaTScan™, an infinite number of cluster circles are generated with a minimum size diameter set to 300 m, the approximate size of the future clusters. A maximum value will initially be set to 1 km and then altered as a function of the number and type (hot/cold) of clusters detected. SaTScan™ identifies hot or cold spots that represent either more or less than the expected dengue cases compared with the entire study area. SaTScan™ then calculates the relative risk of observed hot and coldspots, and statistical significance of these spots is determined using a likelihood ratio test. Only spots with no geographical overlap will be accepted. Areas that are neither hotspots nor coldspots will be classified as intermediate. Hot and cold spots will contain a variable number of *purok*s and it is possible, and indeed likely, that hot and cold spots vary year to year. Therefore, *purok*s will be classified by annual similarity, in other words year 1 in hotspot, year 2 not, year 3 coldspot, and so on. In this way *purok*s can be classified into a risk designation and matched for treatment and control site selection.

#### Sero-conversion

Sero-conversion of individuals to DENV will be analysed yearly using three saliva samples taken from each individual during each year (six total samples per volunteer). Because there are repeated measures from the same individual, sero-conversion is analysed using a generalised linear mixed model (GLMM) with ‘individual’ as a random factor. We will also include cluster ID, school, and occurrence (yes/no) of any public health mosquito control intervention performed in the cluster, and when, as random factors. Sero-conversion will be considered both as 1) a four-fold titre rise in DENV IgG optical density (OD) from baseline to post-treatment (year 1 or 2) samples, thus classifying individuals into yes/no (Y/N) sero-conversion, and 2) the actual change in OD without the restrictive definition of a four-fold increase. This double analysis will enable assessment of change in IgG OD beyond that using the classical threshold of an arbitrary four-fold increase. The Y/N IgG OD analysis will use logistic regression and the latter OD measure log-linear regression. Time (Post1 and Post2 for Y/N logistic regression; Pre-, Post1, and Post2 for log-linear regression), treatment (intervention Y/N), age (continuous), sex (male/female), vaccination status (0, 1, 2, 3 inoculation categories), and site (*purok*) risk (baseline seropositive prevalence rate) will be the explanatory variables. All analyses will be performed using Genstat version 15 [41].

#### Adult mosquito spatial distributions (relative densities) using GATs

Weekly adult mosquito catches will be analysed by fitting a GLMM with the response variable as mosquitoes/trap/week, time (week), and intervention (Y/N) as fixed effects, and cluster ID and cluster risk designation as random effects. In addition, we will include occurrence of any public health mosquito control intervention performed in the cluster by counting the numbers of days during the 2 month inter-saliva sampling period when mosquito numbers could have been affected by such a control intervention (up to 14 days maximum post-control) as a random factor. We will test for over-dispersion in the response variable and, if it exists, an over-dispersion parameter will be estimated and applied. Otherwise, a Poisson distribution will be assumed. Two models will be constructed, one for each *Aedes* dengue vector species.

Additionally, a second series of analyses involving the distance of GATs from the nearest In2Care® trap will be analysed using log-linear regression, fitting the actual Euclidean distance in metres as a continuous variable. Because the extent to which an area is built up can impede mosquito flight (i.e. barriers), each cluster will be categorised into an urban typology (categorical building density) and this will be fitted as a factor in the analyses.

### Data and trial monitoring

In the context of the Ecomore 2 project (*www.ecomore.org/*), of which this trial is part, a Steering Committee composed of five independent individuals has been created. Their role is to: 1) monitor the progress of all the projects towards their objectives, targeted results, and identified milestones/indicators. Monitoring will include annual presentations made by the Partners and the coordination team to review status and progress of the various components and as well as field visits, if relevant; 2) discuss and validate the proposals, recommendations, and guidelines released by the Project Coordinator, the Partners, or the Scientific Advisors; 3) ensure that recommendations/requests issued by the Steering Committee and validated by the Partners through the acceptance of the Steering Committee minutes have effectively been translated into action in the subsequent months; and 4) decide, if necessary, adjustments to the activities programme and original schedules and provide corrective actions on the methodology. All modifications must be clearly stated and formally accepted by the Consortium members (Project coordinator and partners in each participating country).

### Dissemination policy and access to data

Results will be communicated to trial participants through general dissemination meetings and language-adapted leaflets in Tagalog (the national language of the Philippines). Scientific publishing will be in open access, peer-reviewed journals and involve all scientific collaborators, the authors of this manuscript, and others as appropriate. The final results will be presented following the CONSORT 2010 statement and the extension to cluster randomised trials [42]. This study protocol follows the recommendations outlined in SPIRIT (Standard Protocol Items: Recommendations for Clinical Trials) (Additional file 4) and the minimum trial registration information of WHO (Additional file 5). The trial is also registered with International Standard Registered Clinical/Social Study Number (ISRCTN). After the project has officially ended, access to the fully anonymised trial dataset will be made available through the Dryad Digital Repository and all protocols and samples of unfilled consent/information forms and case report forms will be freely available from the authors.

### Archiving

The following documents will be archived for 15 years following the completion of the study: signed research protocol by all research partners (an original kept with each of the partners and an original of all partner signatures with the promoter of the research); consents/non-opposition signed form (original only); observation book/data collection medium (original with initiator of the research and copied to the investigators and/or other collaborators); and final report of the study and formal publications.

## Discussion

It is commonly accepted that an integrated approach to vector control is required to be effective in reducing transmission risk. The WHO Global Strategic Framework for Integrated Vector Management (IVM) recommends a range of interventions in combination to increase impact [43]. Currently, the majority of countries adhere, at least as published policy, to WHO recommendations on mosquito control including source reduction (environmental hygiene and community-based clean-up campaigns), larvicidal treatment of water storage containers, and peridomestic space spraying of insecticides around homes of recent dengue cases [44]. However, there is currently no definitive evidence that these measures have any demonstrable effect on reducing dengue transmission [11, 24, 25].

This efficacy trial of the first commercial product using the AD approach will be highly informative as to the general utility of AD in reducing not only vector mosquito densities but, more importantly, actually reducing the risk of dengue infection. The AD technique may complement typical source reduction practices and larviciding campaigns by more efficiently targeting the most productive containers upon which the female mosquito fixes and contaminates the larval habitat, and importantly those sites beyond access for human manual treatment. AD is potentially a more effective intervention than adult mosquito lethal traps because its impact is amplified between the dissemination devices and the larval habitats, in other words a small number of devices can contaminate a much wider number of habitats [12, 16]. Despite some evidence that PPF disseminated by AD can be effective [12, 15, 23], there remain several obstacles to be overcome. Firstly, the AD devices need to attract sufficient mosquitoes and thus depend not only on the local abundance of mosquitoes but also on the attractiveness of the AD device in the face of other competitive sites. Secondly, studies in urban areas have shown that topography is important for the movement of mosquitoes (e.g. solid structure barriers impeding dissemination) [45]. This implies that AD devices may have to be deployed at very high and operationally impracticable densities. Furthermore, the In2Care® recommendation in areas having high numbers of potential alternative larval habitats is 1 device every 400 m^2^, the equivalent to one trap per 20 m. This high trap density is unlikely to be feasible or even welcome by a community. Thus, there is need for the development of more operationally useful and practical strategies for incorporation of AD approaches into larger, integrated mosquito control programmes. Finally, as with all insecticides, the risk of resistance developing against PPF is a real possibility; therefore, resistance management strategies are required to mitigate this threat.

The evolution of resistance to PPF has been well documented for agricultural pests, most notably for the cotton whitefly *Bemisia tabaci* where PPF resistance became so high in some areas of Israel that it was discontinued [46]. Learning from this unfortunate outcome, an insecticide resistance management (IRM) programme was implemented in the US with recommendations for early season, single application, threshold-based treatments of PPF, coupled with a later season application of broad-spectrum insecticides [47]. Metabolic resistance in both housefly and whitefly to PPF was attributed to mutations in enzyme activities of cytochrome P450 mono-oxygenases and glutathione S-transferases [48, 49], but resulting in a fitness cost that may have helped retard the development of resistance under field conditions when IRM is applied [46, 49]. Using an alternating application of several insecticides with different physiological modes of action has long been a strategy to combat resistance of insects to chemical control agents. The addition of the slower-acting entomopathogenic fungus against adult mosquitoes may contribute to a reduction in the rate of selection pressure for resistance against PPF, in addition to its lethal and pre-lethal effects on the mosquito. The fungus has been shown to counter the effect of insecticide resistance in anopheline mosquitoes [50, 51]. The extent to which resistance can develop against entomopathogenic fungi remains uncertain; whilst it can be selected for, the associated fitness cost to the insect suggests that it is less likely to arise in field settings [52–54]. Indeed, the slow action of the fungus potentially makes it ‘evolution-proof’, as its lethal effect occurs primarily in older mosquitoes that have had the opportunity to breed [55]. The temporal requirement of evolution-proof insecticides is to allow mosquitoes time to develop and oviposit viable eggs but prevent them from transmitting pathogens. Insofar as DENV takes approximately 10 days incubation time from imbibing an infective bloodmeal to being transmitted to a susceptible host, this enables implementation of a slower-acting insecticidal agent whilst reducing onward DENV transmission. This balance is, however, fine and predicated on the circumstances. In conclusion, a resistance management programme should be envisaged if AD mosquito traps prove effective.

Whilst the epidemiological efficacy of the new tool is fundamental, community involvement and optimising strategies for the public health sector can greatly enhance the utility and success of an intervention. In addition to combining different mosquito control tools, the WHO IVM strategy emphasises the necessity to consider the societal impact, public opinion, and awareness in the viability and acceptability of any intervention strategy [56]. The IVM approach also emphasises the importance of strengthening the public and private sectors involved in decision-making and implementation of mosquito control strategies. Carrying out the trial in close collaboration with the local public health sector and included communities is an important step in enabling local support and continuity for vector control.

Surveillance is also an essential component of dengue control because it provides spatial-temporal information on the number and distribution of disease cases and the relative intensity of transmission. Reliable and timely surveillance data allow programmes to target interventions appropriately and to respond to outbreaks quickly. However, integrated epidemiological, entomological, and intervention data are often unavailable and are time-consuming to collate and analyse. This poses a significant challenge to dengue control because the most effective control programmes will likely utilise several complementary interventions. The public health sector is a domain under continuous change and development, and novel methods for improving capacity are increasingly available. Emerging information technologies are improving our capacity to predict, prevent, and control vector-borne and other infectious diseases [57]. Data-rich epidemiological studies and robust surveillance programmes will benefit enormously from such technological advances that will enable rapid collection and analysis of standardised disease and vector data. Continuous surveillance, monitoring, and evaluation using real-time data enables vector-borne disease control programmes to rapidly identify disease occurrence, monitor specific intervention effectiveness, and plan appropriate interventions.

In conclusion, there is a suite of technological tools for the development of more up-to-date approaches to vector mosquito control to reduce viral pathogens transmitted by *Aedes aegypti* and similar species. However, new tools require validation under field conditions before optimisation for wide-scale and routine application. Well-executed evaluations for assisting local public health sectors to achieve technologically advanced management of their mosquito control strategies are a sine qua non for reducing the burden of mosquito-borne disease.

## Study limitations

The major concern of the study will be the dependency on there being sufficient dengue activity during at least one of the study years and that the cluster design is sufficient to account for the recognised spatio-temporal heterogeneity in dengue [58, 59]. To maximise power and somewhat alleviate this concern, sero-conversion rather than dengue case incidence will be used as the measure of epidemiological outcome, capturing the sub-clinical infections that form the majority of dengue infections [60, 61]. Continued compliance of participants during the 2 years may pose a problem as will community acceptance of the In2Care® traps and GAT devices. Considerable effort will be given to continuous dialogue with the community to maintain adherence throughout the study.

## Additional files

Additional file 1: Gravid *Aedes* trap. Courtesy of B. Lefebvre. (JPG 103 kb)

Additional file 2: Assembly of In2Care® trap. Courtesy of S. Boyer. (JPG 234 kb)

Additional file 3: Sticky card with mosquitoes captured in a Gravid *Aedes* Trap. Courtesy of O. Telle. (JPG 97 kb)

Additional file 4: SPIRIT checklist. (DOCX 66 kb)

Additional file 5: WHO Trial Registration Data Set (Version 1.3). (DOCX 24 kb)

Additional file 6: Information and consent forms. (DOCX 198 kb)

## Abbreviations

AD: Auto-dissemination
BHW: *Barangay* health worker
CAS: Chemical Abstracts Service
DDMS+: Disease Data Management System
DENV: Dengue virus
DoH: Department of Health
ELISA: Enzyme-linked immunosorbent assay
GAT: Gravid *Aedes* trap
GLMM: Generalised linear mixed model
Ig: Immunoglobulin
IRM: Insecticide resistance management
IVM: Integrated Vector Management
OD: Optical density
PCRT: Parallel, two-armed cluster randomised trial
PIDSR: Philippines Integrated Disease Surveillance and Response
PPF: Pyriproxyfen
RITM: Research Institute for Tropical Medicine
SPIRIT: Standard Protocol Items: Recommendations for Clinical Trials
WHO: World Health Organization
