# Supplementary figures and images for: Efficacy of the In2Care® auto-dissemination device for reducing dengue transmission: study protocol for a parallel, two-armed cluster randomised trial in the Philippines
Source: Trials. 2019 May 14;20:269. doi: 10.1186/s13063-019-3376-6 (PMC6518692; doi:10.1186/s13063-019-3376-6)

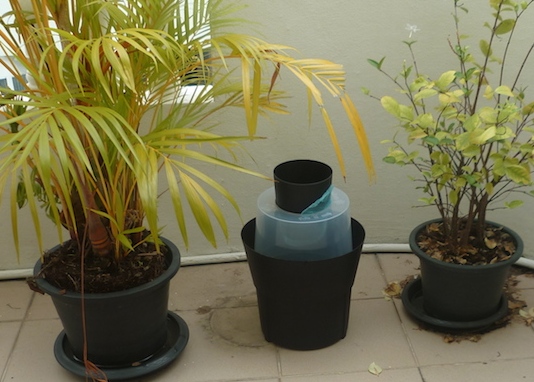

Supplement: Supplementary file 1 — Gravid Aedes trap. Courtesy of B. Lefebvre. (JPG 103 kb) [file 13063_2019_3376_MOESM1_ESM.jpg]

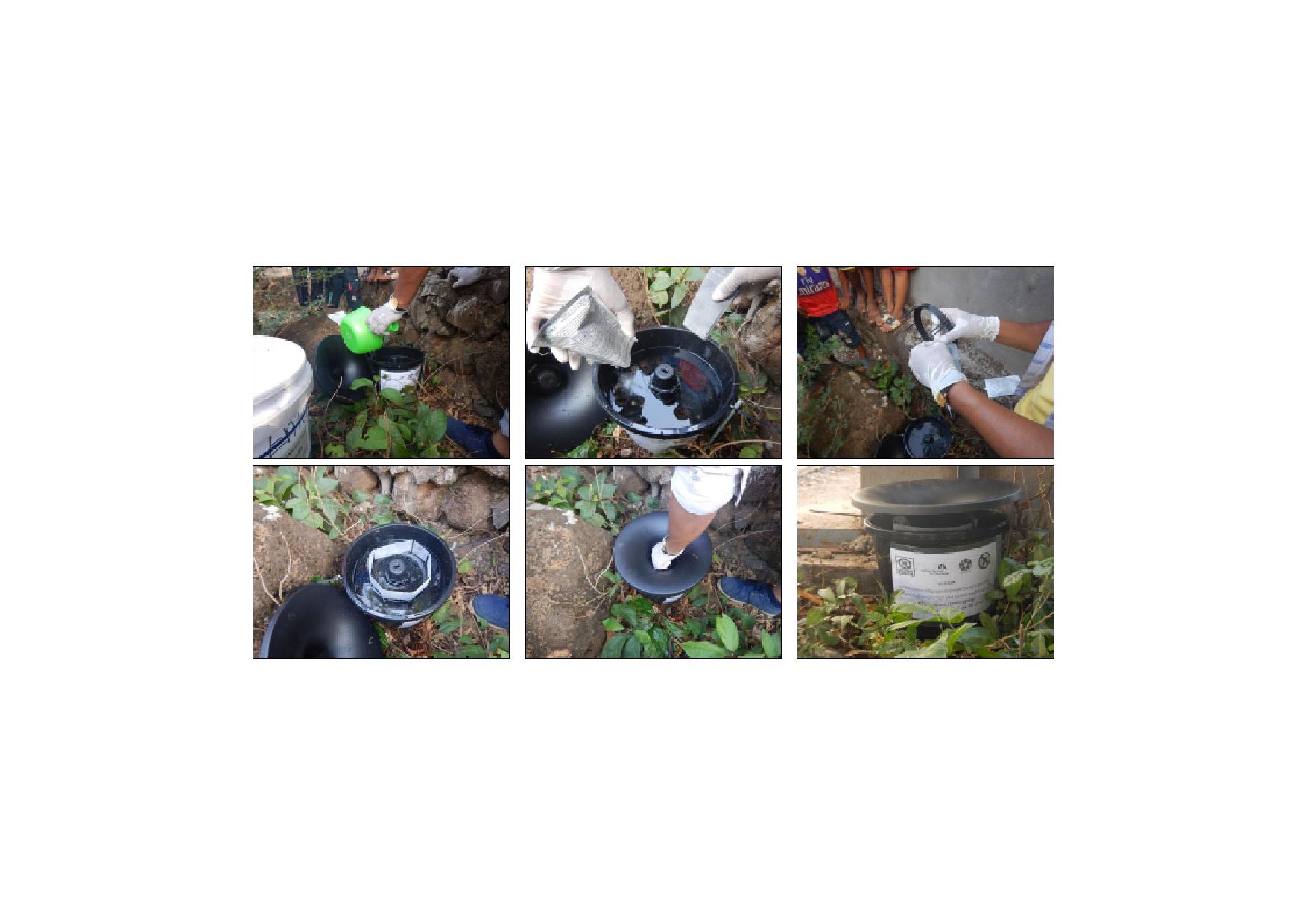

Supplement: Supplementary file 2 — Assembly of In2Care® trap. Courtesy of S. Boyer. (JPG 234 kb) [file 13063_2019_3376_MOESM2_ESM.jpg]

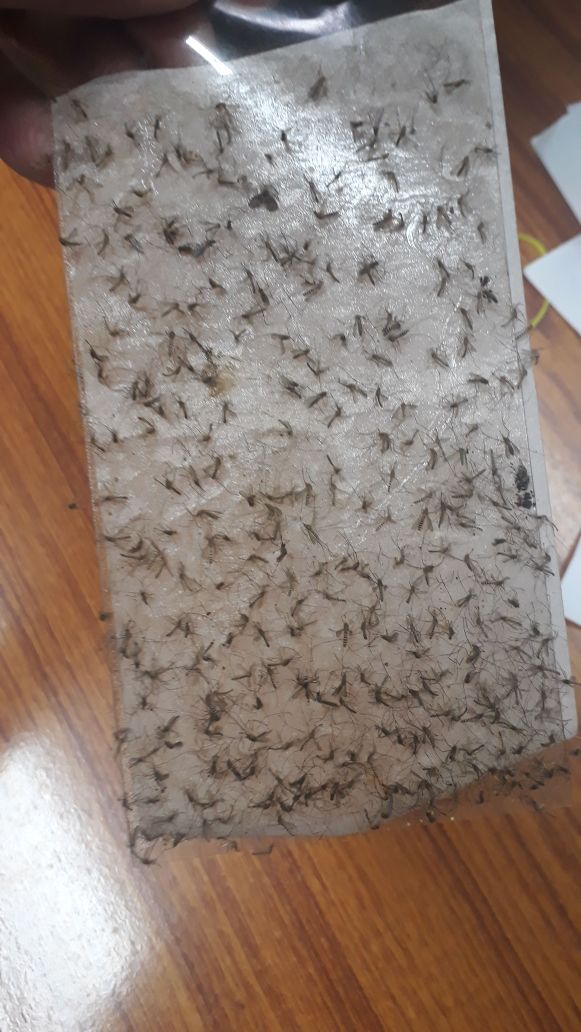

Supplement: Supplementary file 3 — Sticky card with mosquitoes captured in a Gravid Aedes Trap. Courtesy of O. Telle. (JPG 97 kb) [file 13063_2019_3376_MOESM3_ESM.jpg]
