# Supplementary material for: Efficacy of the In2Care® auto-dissemination device for reducing dengue transmission: study protocol for a parallel, two-armed cluster randomised trial in the Philippines
Source: Trials. 2019 May 14;20:269. doi: 10.1186/s13063-019-3376-6 (PMC6518692; doi:10.1186/s13063-019-3376-6)
Supplement: Supplementary file 5 — WHO Trial Registration Data Set (Version 1.3). (DOCX 24 kb) [file 13063_2019_3376_MOESM5_ESM.docx]

**Additional file 5. WHO Trial Registration Data Set (Version 1.3) based on** [**http://www.who.int/ictrp/network/trds/en/**](http://www.who.int/ictrp/network/trds/en/)

| **#** | **Data category** | **Information** |
| --- | --- | --- |
| 1 | Primary Registry and Trial Identifying Number | ISRCTN Registry, Pending |
| 2 | Date of Registration in Primary Registry | 4 December 2018 |
| 3 | Secondary Identifying Numbers | IP No. 2018-03; RITM No. 2018-09 |
| 4 | Source(s) of Monetary or Material Support | Agence Française de Développement |
| 5 | Primary Sponsor | Institut Pasteur, Paris, France |
| 6 | Secondary Sponsor(s) | - |
| 7 | Contact for Public Queries | REP [rpaul@pasteur.fr] |
| 8 | Contact for Scientific Queries | REP [rpaul@pasteur.fr] |
| 9 | Public Title | - |
| 10 | Scientific Title | Efficacy of the In2Care® auto-dissemination device for reducing dengue transmission: study protocol for a parallel two-armed cluster randomised trial in the Philippines |
| 11 | Countries of Recruitment | The Philippines |
| 12 | Health Condition(s) or Problem(s) Studied | Dengue |
| 13 | Intervention(s) | Active comparator: Pyriproxyfen+Beauvaria in the In2Care mosquito trap for four months per year for 2 years.  Control: Normal governmental dengue control activities |
| 14 | Key Inclusion and Exclusion Criteria | Inclusion Criteria (Information and Consent forms as attached documents)  • 6-16 year-old, male and female individuals  • Parent/guardian informed consent for the child’s participation in the study  • For children > 7-year-old, required assent for his/her participation in the study  Non-inclusion Criteria  • Children with known concomitant pathology(ies) at the time of the consent as indicated by parent/guardian |
| 15 | Study Type | Interventional  Study design: The study is a parallel two-armed cluster randomised trial (PCRT) evaluating the efficacy of the In2Care® Mosquito Trap for reducing incidence of dengue infection over a 2-year period using 4 consecutive months of treatment each year.  Primary purpose: Prevention |
| 16 | Date of First Enrollment | February 2018 |
| 17 | Sample Size | 2 strata, 23 clusters per strata, 100 children per cluster: 2x23x100=4600. |
| 18 | Recruitment Status | Recruiting starts in February |
| 19 | Primary Outcome(s) | The principle evaluation criterion is the epidemiological efficacy read-out: dengue infection sero-conversion. |
| 20 | Key Secondary Outcomes | The secondary evaluation criterion is the entomological read-out. Using Gravid Aedes traps we will measure adult mosquito numbers. |
| 21 | Ethics Review | This trial protocol (version 2, 21 May 2018) was approved by the Research Institute for Tropical Medicine Institutional Review Board (Reference No. 2018-09, 1 June 2018) and the Institut Pasteur Institutional Review Board (Reference No. 2018-03, 14 June 2018). |
| 22 | Completion date | March 2021 (expected) |
| 23 | Summary Results | Not started yet |
| 24 | IPD sharing statement | Undecided |
