# Supplementary material for: Efficacy of the In2Care® auto-dissemination device for reducing dengue transmission: study protocol for a parallel, two-armed cluster randomised trial in the Philippines
Source: Trials. 2019 May 14;20:269. doi: 10.1186/s13063-019-3376-6 (PMC6518692; doi:10.1186/s13063-019-3376-6)
Supplement: Supplementary file 6 — Information and consent forms. (DOCX 198 kb) [file 13063_2019_3376_MOESM6_ESM.docx]

**MOSQUITO CONTROL IN A VACCINE SITE**

**(Research Institute for Tropical Medicine)**

Version Date: **15 May 2018**

Principal Investigator: **Richard Paul**

Institut Pastur, Paris, France

Co-Investigator: **Ferdinand V. Salazar, DAP&E, PhD**

Research Institute for Tropical Medicine

**Edward Thomsen**

Liverpool School of Tropical Medicine, United Kingdom

Sponsor: Institut Pasteur, Paris, France

*You are being asked to participate and authorize your child’s participation in a research study on dengue fever performed in collaboration with colleagues from the Philippines, France and the United Kingdom. The purpose of this consent form is to give you information necessary to help you decide whether or not to be in the study. Please read the form carefully. You may ask questions about the research or this form that is not clear.*

**Introduction.** Dengue fever is a very common disease in the Philippines and there are more than 100 million cases recorded in the world every year. Dengue fever is a disease caused by a virus that is transmitted by mosquito bites. In this study we will place mosquito traps containing insecticide around the house. The insecticide in the trap will kill the mosquito leading to the decrease of the mosquito population that can result to a decrease of people at risk of being infected with dengue. As a consequence of the insecticide, the number of mosquitoes will decrease and this could contribute to the decrease of dengue infected people. This insecticide is widely used in agriculture and only affects insects. It has no effect at all on vertebrates including humans, dogs, cats, birds, lizards, frogs, fish etc. If we show that these traps decrease dengue then this could be used as a mosquito control method to reduce dengue here and throughout the Philippines.

**Study Procedures.** In this study we will place commercial mosquito traps outside your houses. There are two types of mosquito traps to be installed: (1) the bucket like traps catch mosquitoes and measure how many mosquitoes there are (see Figure 1, left); (2) the other traps attract mosquitoes that then fly off with insecticide on their legs and deposit it in places where they lay their eggs in places that hold water, such as used tires, flower pots, empty cans (see Figure 1, right). Mosquitoes trapped will be collected and brought to the laboratory for identification.


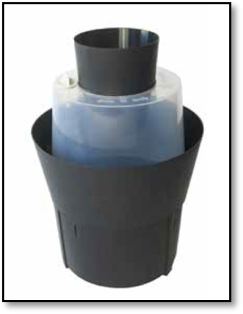

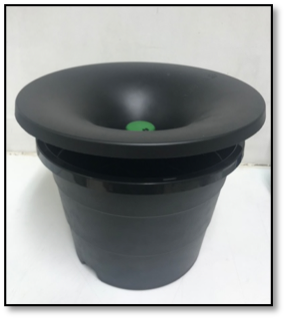


Figure 1. Mosquito traps: left - Gravid Aedes Trap (GAT), right – In2Care Trap

To assess whether the mosquito traps work, we will ask your children (only those between 4 to 15 years old) to donate saliva to test whether or not they have been infected by the dengue virus. For this we would like the child to spit into the container provided. Once saliva has been tested it will be disposed properly. The result of the tests will be given back to you. Individual participants and their families will be informed of any new information pertinent to the study arising during the study.

The two-year study will place mosquito traps for 4 months and serviced on a weekly basis. At the time of trap installation, saliva will also be collected. Saliva collection will be repeated 2 months and 4 months after initial collection.

In the Philippines, this research study has been approved by the competent health authorities and ethics committees.

**Risks, Harm, Discomfort.** There is no harm in participating in the study. The traps that will be installed will not serve as breeding site, instead it will act as a tool to kill adult flying mosquitoes or as an “auto-dissemination” tool providing an effective kill of mosquito larvae in breeding sites surrounding the trap.

**Benefits, Compensation.** You will incur no cost nor will you be paid for joining the study but the research findings will help the researchers in making recommendations to control and prevent dengue. Significant information on the disease and vector gathered in the study will be communicated to you.

**Voluntary Participation & Withdrawal.** You have the right to decide whether you want to participate or not. If you decide to participate in the study, we will ask you to sign a consent form. You may withdraw your participation in the study after you have agreed to participate, at any time without any penalty.

**Confidentiality & Access to Participants Records.** We will keep the information we get from you as private. Identification number will be assigned to your child to ensure confidentiality. The correspondence between your child’s name and the number will figure in a matching list which will be kept privately until the return of the final tests. This matching list will be then destroyed and your data will be completely anonymous afterwards. Your child’s name will not be mentioned in any paper or speech about the study. There will be compliance of the study to the Republic Act No. 10173 (Philippines Data Privacy act) will full protection of individual’s personnel information by coding and secure data storage, with no dissemination of this information outside of the study context. Upon completion of the study this information will be destroyed. Individual participants can ask for their information to be removed and destroyed at any time during the study.

If you have any other questions about the study please ask the following persons:

| RITM - Department of Medical Entomology: (02) 807-2628 to 32 local 603 | | |
| --- | --- | --- |
| **Dr. Ferdinand V. Salazar**  09175465796 (Globe) | **Ariza Minelle A. Aguila**  09176513244 (Globe) | **Jason R. Angeles**  09177213831 (Globe) |

For further questions and clarifications on your rights as research participants, you may contact:

| RITM - Institutional Review Board: (02) 807-2628 to 32 local 418 | |
| --- | --- |
| **Dr. Veronica Tallo** (Chair) | |
| 09060937522 (Globe) | 09294480180 (Smart) |

**MOSQUITO CONTROL IN A VACCINE SITE**

- 4-6 years old
- 7-15 years old

# WRITTEN PARENTAL CONSENT FORM

Version date: 15 May 2018

I, , parent/guardian of , ,

(Parent/Guardian’s Name) (Child’s Name) (Age)

was invited to participate in the research to evaluate the epidemiological and entomological efficacy of a mosquito trap and allowing my child to provide saliva to test infection to dengue virus.

I have heard and read the information sheet and the consent form in the presence of an independent witness. I also have been given explanation concerning the purpose/methodology of the study, possible risk and benefit that may occur to me upon participation of the study, up to my satisfaction and understanding.

Please mark the sign × in front of the statement you understand and you agree with.

I have been fully informed of the possible risks and benefits for taking part in this study.

I allow placement of mosquito traps outside my house.

I authorize the above-mentioned child to take part in this study on the dengue fever. I agree that the collected saliva samples may be tested for exposure to dengue on these samples to further the understanding of our fight against this disease. I understand that once the saliva has been tested it will be destroyed.

I agree that the child’s personal de-identified or anonymous data can be accessible to and analysed by local and foreigner scientists collaborating in this research program

I have read and understood all statements in this consent form before signing my name.

Name of Parent/Guardian Signature Date

Name of Investigator Signature Date

Thumb mark for illiterate participants

Witness testimony:

The objective, procedures, risks and benefits of the study have been explained to the participant and he/she was encouraged to ask questions for clarifications. The participant was also advised that he/she may not participate in the research study and may withdraw their participation at any time. This would not affect him/her or their family. The participant was also informed that his/her identification shall be kept confidential and shall be known only to the researchers.

Name of Witness Signature Date

**MOSQUITO CONTROL IN A VACCINE SITE**

For participants

7-15 years old

# WRITTEN ASSENT FORM

Version date: 15 May 2018

I, , , was invited to participate in the research to evaluate

(Name) (Age)

the epidemiological and entomological efficacy of a mosquito trap and providing saliva to test infection to dengue virus.

I have heard and read the information sheet and the consent form in the presence of an independent witness. I also have been given explanation concerning the purpose/methodology of the study, possible risk and benefit that may occur to me upon participation of the study, up to my satisfaction and understanding.

I have read and understood all statements in this consent form before signing my name.

Name Signature Date

Name of Investigator Signature Date

Thumb mark for illiterate participants

Witness testimony:

The objective, procedures, risks and benefits of the study have been explained to the participant and he/she was encouraged to ask questions for clarifications. The participant was also advised that he/she may not participate in the research study and may withdraw their participation at any time. This would not affect him/her or their family. The participant was also informed that his/her identification shall be kept confidential and shall be known only to the researchers.

Name of Witness Signature Date
